# A framework for modeling county-level COVID-19 transmission

**DOI:** 10.3389/fpubh.2025.1608360

**Published:** 2025-08-05

**Authors:** Yida Bao, Iris Huang, Qi Li, Zheng Zhang, Yuan Xing, Dongfang Hou, Jiafeng Ye

**Affiliations:** ^1^Department of Mathematics, Statistics and Computer Science, University of Wisconsin-Stout, Menomonie, WI, United States; ^2^Inglemoor High School, Kenmore, WA, United States; ^3^Department of Mathematics and Computer Science, Fisk University, Nashville, TN, United States; ^4^Department of Computer Science and Information Systems, Murray State University, Murray, KY, United States; ^5^Research Centre of Light Alloy Net Forming, Shanghai Jiao Tong University, Shanghai, China

**Keywords:** COVID-19, county-level analysis, spatial dependence, multilevel modeling, geographically weighted regression, Moran

## Abstract

This study examines COVID-19 transmission across 3,142 U.S. counties using a truncated dataset from March to September 2020. County-level factors include demographics, socioeconomic status, environmental conditions, and mobility patterns. Ordinary Least Squares regression establishes a baseline for analyzing COVID-19 confirm case counts for each county. We then use Moran's *I* to evaluate spatial clustering, prompting Spatial Autoregressive and Spatial Error Models when autocorrelation is significant. Notably, spatial models outperform the Ordinary Least Squares approach—*R*^2^ rises from 0.4849 with Ordinary Least Squares to 0.6846 under Spatial Error Model, while RMSE decreases from 2.0891 to 1.642—demonstrating improved fit and more accurate spatial transmission dynamics. A multilevel framework further explores state-level policy variations. Finally, Geographically Weighted Regression captures spatial non-stationarity by mapping local coefficient differences; we visualized temperature, precipitation, and other key variables—revealing precipitation peaks near 110° W in the Southeast and Northeast and strong sensitivity to temperature. This integrated sequence of methods provides a comprehensive lens for studying epidemiological phenomena. While certain findings align with established research, other variables reveal unexpected patterns. The proposed framework offers a robust template for future investigations where spatial dependence and policy heterogeneity warrant close examination.

## 1 Introduction

The coronavirus disease 2019 (COVID-19) pandemic has caused an unprecedented global health crisis. The United States has been especially hard-hit; by June 30, 2020 it accounted for ~2.6 million confirmed cases and 127,000 deaths (1). These impacts have not been uniform across communities. On the contrary, COVID-19 outcomes exhibit striking geographic disparities in the U.S. For example, as of late 2020, a U.S. county at the 75th percentile of COVID-19 cases per capita had about double the cases of a county at the 25th percentile (2). Such heterogeneity suggests that underlying county-level factors from demographics and health resources to social behaviors play a crucial role in shaping the spread and severity of COVID-19 (3). Understanding these local drivers is essential for designing targeted public health interventions and informed policy responses.

County-level studies of COVID-19 are therefore of great significance. A growing body of research has begun to explore how county characteristics are associated with COVID-19 incidence. These analyses enable researchers to capture fine-grained variations that would be masked in state or national averages. Killeen et al. (4), for instance, compiled a comprehensive county-level COVID-19 dataset integrating over 300 variables encompassing population demographics, socioeconomic indicators, healthcare capacity, and even mobility patterns. Such rich data resources have facilitated numerous ecological studies. Using county-level data, Pan et al. (1) identified health and social factors linked to COVID-19 mortality across all 3,141 U.S. counties. These findings highlight how pre-existing health disparities and social determinants at the county level can translate into unequal COVID-19 outcomes.

Beyond health and demographic vulnerabilities, researchers have examined a wide array of contextual variables. For example, community mobility and adherence to social distancing have been studied as key determinants of COVID-19 spread. Analyses of cell-phone mobility data indicate that decreases in movement (greater social distancing) are associated with reductions in COVID-19 case growth rates (5). Conversely, counties characterized by lower compliance with distancing tended to have faster viral spread (5). Environmental factors have also received attention: temperature, humidity, and other weather variables were hypothesized to influence viral transmission. Early evidence on meteorological effects was mixed. Some global-scale studies suggested only a minor direct effect of weather on COVID-19 transmission when human factors were not accounted for (6), whereas other studies posited that extremely low humidity and temperature might facilitate spread under certain conditions (7). In the U.S., distinctive local outbreaks underscored the role of specific environmental and occupational settings. Notably, meat and poultry processing plants emerged as infection epicenters in spring 2020 (8). These diverse findings from prior research underscore the multifactorial nature of COVID-19 spread at the county level, involving a combination of population attributes, health disparities, policy responses, behavior, and environment.

Most prior studies have employed a single modeling approach (typically a standard regression) for a given outcome, which may not fully account for the complex spatial and hierarchical structure of the data (9). COVID-19 incidence in one county is not independent of neighboring counties due to spatial diffusion of the virus and regional similarities; Counties are nested within states, and share state-level policies and resources (10). Indeed, spatial analyses have revealed significant clustering of COVID-19 outcomes. Mollalo et al. (10), for example, showed that incorporating spatial autocorrelation via spatial lag and error models greatly improved the fit of an ordinary least squares model for county-level incidence (10). However, even after accounting for global spatial dependence, substantial local variation remained, and a Geographically Weighted Regression model captured those local non-stationarities far better, explaining around 68% of the variance in COVID-19 incidence compared to only 30% by a global Ordinary Least Squares. These results suggest that relationships between predictors and COVID-19 outcomes can vary across space, and no single modeling strategy is sufficient to unveil the full picture.

There is value in comparing what each method (classical Ordinary Least Squares, spatial models, multilevel models, and local regression) contributes to our understanding within a unified study. In light of these gaps, the present study provides a comprehensive statistical analysis of county-level COVID-19 confirmed cases, aggregated from March through September 2020 into a single cross-sectional dataset. First, we establish a baseline with Ordinary Least Squares regression to identify general associations under standard assumptions. We then employ Spatial Autoregressive Models to explicitly model spatial diffusion effects (i.e., the influence of cases in neighboring counties) and Spatial Error models to account for spatially autocorrelated error structures (unobserved spatially clustered factors). These spatial models help ensure that residual spatial dependence is addressed, improving estimation accuracy and inference. Next, we implement a multilevel model (Hierarchical Linear model) treating counties as nested within higher-level units (such as states or regions), which allows us to control for unmeasured state-level influences and borrow strength across counties with similar contexts. Finally, we apply Geographically Weighted Regression as a local modeling approach to explore spatial heterogeneity in the relationships: Geographically Weighted Regression relaxes the assumption of spatially constant coefficients, revealing how the influence of a predictor may differ in magnitude or direction from one region to another. By comparing results across these methods, we can cross-validate findings and gain a nuanced understanding of both global and local patterns.

In summary, this study contributes a multi-method investigation of county-level COVID-19 dynamics during the first six months of the pandemic. This comprehensive framework allows us to address issues of spatial autocorrelation, hierarchical data structure, and non-stationarity simultaneously challenges that, if unaddressed, can lead to biased or incomplete conclusions. The findings not only deepen our understanding of the early pandemic drivers at a fine spatial scale, but also provide practical insights for public health officials. Overall, our statistics-based framework demonstrates a template for rigorously analyzing public health data with complex spatial dependencies, and it advances the literature by uniting multiple analytical perspectives to paint a more complete picture of COVID-19's spread across the American landscape.

## 2 Data introduction

We conduct an analysis of a county-level dataset detailing the COVID-19 outbreak in the United States. The dataset (11) provides reliable daily counts of confirmed cases for a wide geographical area, thereby facilitating robust predictive modeling and in-depth epidemiological analyses. The data span from January 22, 2020, to September 16, 2020—a critical interval that captures the early evolution of the pandemic and the impact of various predictive features (11). Although the national series starts on 22 January 2020, we truncate it to 1 March–16 September 2020 for all analyses because the vast majority (>99%) of counties reported zero cumulative cases prior to March, and the sparse early counts add noise but no information to the spatial models. In addition to the daily case counts, the dataset contains a wide range of county-level features. These include demographic variables, socioeconomic indicators, health metrics, and infrastructure measures. Geographic information is also provided for each county. Our approach is designed to capture both general and spatial patterns in the data. We begin by exploring overall relationships among the variables. We then assess potential dependencies and variations across space. We also account for differences at multiple hierarchical levels.

Temperature and precipitation records had numerous missing entries, so we filled these gaps by incorporating annual summary data sourced from the National Centers for Environmental Information (12).

### 2.1 Data cleaning

The dataset covers 3,142 U.S. counties. Daily variables include confirmed COVID-19 cases, social distancing grade, temperature, and precipitation. These data include demographic characteristics (e.g., female percent, population density, immigrant student ratio), socioeconomic status (e.g., gdp per capita, median household income, political party), health indicators (e.g., percent diabetes, percent smokers), and infrastructure measures (e.g., hospital beds ratio, ventilator capacity ratio).

Table 1 provides an overview of the variables used in the analysis. The first column indicates the data type (e.g., Continuous, Percent, Binary), the second column lists the abbreviated variable names, and the third column offers a brief description of each variable.

**Table 1:** Variable descriptions.

**Data type**	**Variable name**	**Description**
	cases_per_10k	Confirmed COVID-19 cases in each county
	temp	Average temperature (°F)
	precip	Average precipitation (mm)
	daily_test	Daily tests performed (count)
	pop_dens	Population density (people/km^2^)
	area	County area (km^2^)
Continuous	vent_cap	Ventilator capacity ratio
	tot_coll_pop	Total college population (count)
	ap_dist	Distance to nearest airport (km)
	pass_load	Passenger load ratio
	gdp_pc	GDP per capita (USD)
	med_income	Median household income (USD)
	hosp_beds	Hospital beds ratio (per 1,000 people)
	old	Proportion of older population (e.g., 65+)
	female_pct	Proportion of female population
	less_HS	Proportion with < high school diploma
	some_coll	Proportion with some college or higher
Percent	diabetes_pct	Proportion with diabetes
	relig_cong	Religious congregation ratio
	insured_pct	Proportion of insured population
	immig_stud	Proportion of immigrant students
	smokers_pct	Proportion of smokers
Ordinal	SD_travel	Travel distance based social distancing grade 5 best to 1 worst.
	meat_plant	Meat processing facilities count
Binary	pol_party	Political party preference 0 = republican, 1 = democrat

Although the raw dataset provides daily observations, we require county-level data for our analysis. We aggregated the daily data for each county. Specifically, the cumulative number of confirmed cases and precipitation were computed by summing their daily values. For the remaining daily variables—daily test, daily state test, temperature, precipitation, social distancing total grade, social distancing encounters grade, and social distancing travel distance grade—the mean values were calculated across the study period. Static county-level attributes (e.g., demographic, socioeconomic, and health system indicators) remained unchanged. Each county is represented as a single observation in the final dataset. This structure facilitates statistical modeling and epidemiological assessment. Since Daily data often contain short-term fluctuations and noise. Aggregation reduces random measurement errors and highlights long-term trends (13). This approach aligns the analysis with administrative boundaries, which are relevant for policy decisions. Moreover, it mitigates temporal autocorrelation, allowing the focus to shift to spatial heterogeneity. Such aggregation is common in epidemiological studies that seek to understand broad patterns over time (13).

This study focuses on the primary response variable, which is the number of confirmed cases per 10k people. We deliberately use this ratio to ensure that county population size does not influence the results. This measure is common in epidemiological studies and offers a standardized way to compare infection rates across regions (14).

### 2.2 County-level case rate map

**Figure 1:**
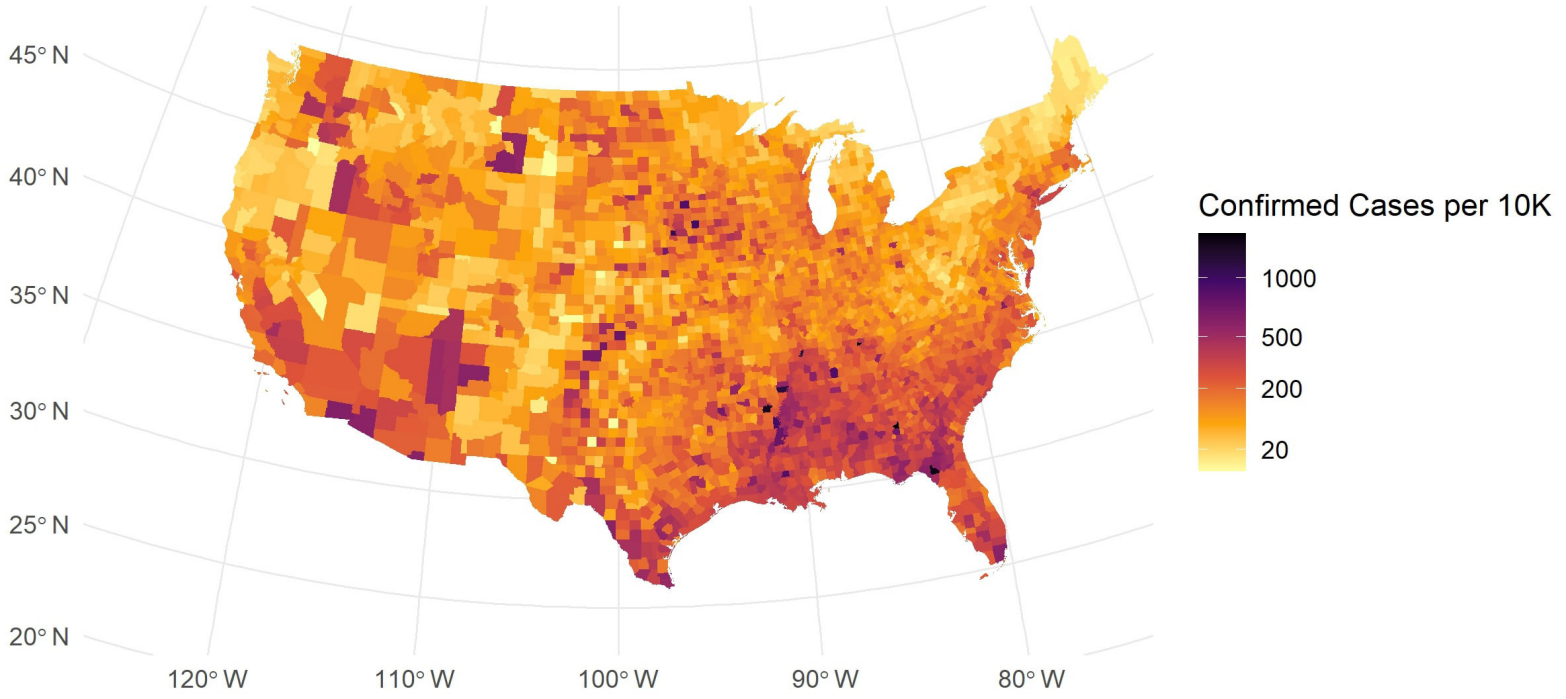
Map of confirmed cases per 10K people in USA Mainland.

For our data description, we incorporated a U.S. map shapefile obtained from the U.S. Census Bureau's mapping files (15). This spatial dataset allows us to explore how COVID-19 spreads in different regions. As shown in Figure 1, the map of confirmed cases per 10k people exhibits a strong spatial pattern.

In Figure 1, while some counties in major urban areas have high confirmed case counts, these are depicted as blue-black points scattered across the country. Many areas in the Southeast and Southwest exhibit elevated infection levels, and some coastal urban centers also show higher densities. By contrast, large portions of the Midwest and parts of the Northeast reveal more moderate or lower rates. These spatial patterns suggest that local demographic, economic, and policy factors may drive substantial regional variation in COVID-19 burden. This spatial disparity in COVID-19 incidence suggests that factors such as regional demographic characteristics, socio-economic conditions, and local policies may play significant roles.

### 2.3 Other variables

**Figure 2:**
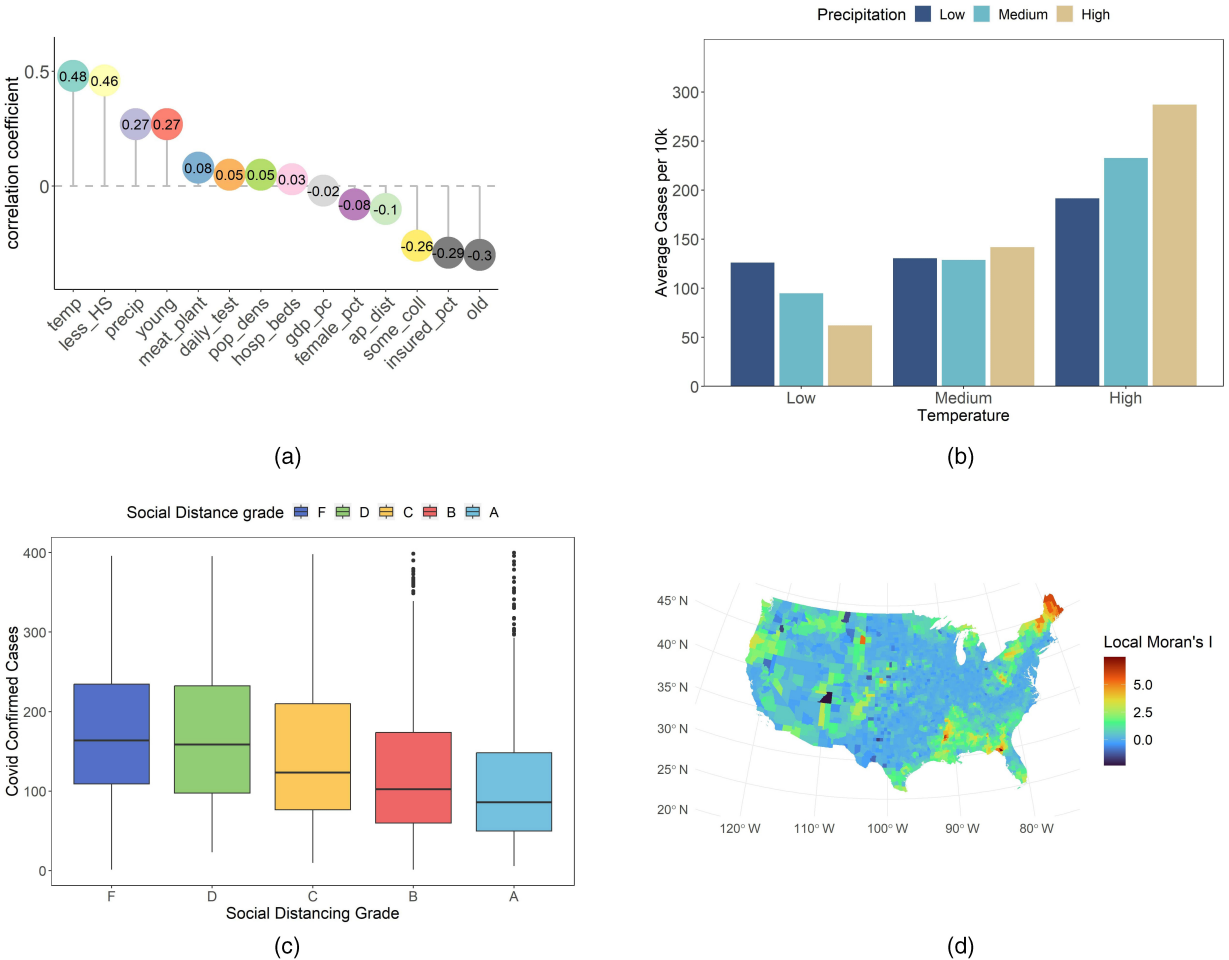
Overview of some case-related variables. **(a)** Correlation of each variable with COVID-19 confirmed cases per 10k residents. **(b)** Average cases per 10k by temperature and precipitation groups. **(c)** COVID-19 confirmed cases per 10k residents across different social distancing grades. **(d)** County-level Local Moran's *I* values for COVID-19 cases per 10k residents.

Let us examine additional variables to uncover further insights. Figure 2a shows a chart of selected key variables, where the dependent variable is cases per 10k. We include variables from several domains: climate, population structure, socio-economic, education, medical and health behavior, and intervention and location. In the chart, the correlations are sorted in descending order—from strong positive values to negative values. Temperature and precipitation show strong positive correlations; similar findings have been reported in previous research (16). The proportion of young individuals also correlates positively with infection rates. In contrast, the percentages of older individuals and insured population, as well as airport distance, correlate negatively with cases per 10k. These observations serve as an initial descriptive overview.

Our analysis continues to focus on the detailed role of climate. We divided temperature and precipitation into low, medium, and high groups by percentile. Based on Figure 2b confirmed cases increase as temperature and precipitation rise. Under low temperature conditions, lower precipitation is linked to higher confirmed cases than higher precipitation. In medium temperature settings, the difference due to precipitation is minimal. Under high temperature conditions, high precipitation is associated with more severe infections compared to low precipitation. In environments with high precipitation, temperature changes yield a greater sensitivity in confirmed cases than in those with low precipitation. These observations may reflect complex interactions between climatic factors and transmission dynamics. Lower confirmed cases in low temperature and high precipitation areas may be linked to reduced outdoor activity. For example, Washington state experienced frequent winter rain and adopted early strict measures (17). New York enhanced public responses after initial outbreaks (17). In contrast, high temperature and high precipitation regions show increased indoor gatherings. Florida residents often meet in air-conditioned spaces (16). Such examples indicate that climatic conditions may alter behavior and viral stability. These examples suggest that climatic conditions may interact with human behavior and virus stability (18, 19).

The social distance grade is derived from Unacast, and is based on mobile location data (20). It is based on three metrics: travel distance, visitation patterns, and human encounters (11). The travel distance metric indicates the percentage reduction in the average distance traveled in each county (11, 21). The visitation patterns metric represents the percentage change in visits to non-essential venues (11, 21). The human encounters metric measures the reduction in encounter density. An encounter is defined as two devices being within a 50-m radius for < 1 h. The total social distance grade is the average score of these metrics (11, 21). Grades were assigned based on the reduction level relative to a pre-COVID-19 baseline. For instance, the social distancing encounters grade is defined as follows: Grade A indicates a reduction of more than 94%; Grade B reflects a reduction between 82 and 94%; Grade C represents a reduction between 74 and 82%; Grade D corresponds to a reduction between 40 and 74%; and Grade F signifies a reduction of < 40% or an increase (11). We use the total social distance grade together with confirmed COVID-19 cases at the county level. Our goal is to explore the link between social distancing performance and infection rates under varied policy backgrounds. We trim the data by removing outliers. In our graphs, points with more than 400 confirmed cases per 10k residents are excluded. This step enhances the clarity of the analysis. The Boxplot in Figure 2c indicates little differences between grades F and D. A systematic trend emerges from grade D through C, B, and A. As the social distance grade improves, confirmed case counts decline. Outbreak severity also diminishes with higher grades. This pattern is observed consistently across various counties. These findings suggest a potential relationship between adherence to social distancing measures and lower infection rates. In addition, the data reflect the impact of current public health policies on individual behavior. Policy interventions that reinforce social distancing are shown to have a measurable influence. These observations provide an initial framework for assessing policy effectiveness and guide future adjustments.

We also analyze county-level COVID-19 cases per 10K using local Moran's *I*. This statistic quantifies spatial autocorrelation and identifies clusters of similar values. It has been widely applied in spatial epidemiology (22) and featured in recent COVID-19 studies (23). High local Moran's *I* values typically denote high–high or low–low clusters. Our approach employs this metric to explore disease spread patterns. In Figure 2d, local Moran's *I* shows notable clustering for COVID-19 cases per 10K. Initial inspection revealed elevated values in the South, Northeast, and northwestern Oregon. Comparing with Figure 1, we may identify low–low clusters in the Northeast and Oregon, and high–high clusters in the South. These results underscore the need to account for spatial dependence in our COVID-19 analysis. The clustering patterns observed at the county level suggest that spatial autocorrelation is a key factor. This evidence provides a robust basis for integrating spatial considerations in our future modeling efforts.

In our work, county-level data on COVID-19 cases and various predictive features have been examined. The dataset was aggregated from daily in the previous sections we provided a concise overview of county-level COVID-19 data with emphasis on climatic factors. Temperature and precipitation groups were highlighted while other variables received limited discussion. The next section presents our methodological framework. We describe the statistical models and analytical techniques used to examine these interactions.

## 3 Methodology

We aim to establish a robust empirical framework by sequentially applying several statistical models, each capturing different characteristics of the pandemic. Throughout our approach, we adopt epidemiologic methods, spatial analysis techniques, and regression models commonly used in public health research (24). Our primary interest lies in understanding spatial dependence rather than forecasting, so we employ the entire dataset without partitioning it into training and testing subsets.

### 3.1 Baseline ordinary least squares

We begin with a baseline Ordinary Least Squares (OLS) model to examine the relationship between relevant explanatory variables and the dependent variable, denoted as *y*_*i*_ = *confirm_case_per_10k* for county *i*. The model assumptions include linearity, homoscedasticity, normality of residuals, and independence of errors.

Due to spatial clustering, county-level COVID-19 data often violate the assumptions of independence and homoscedasticity. The variable *confirm_case_per_10k* exhibits a skewness of 2.54. We apply a Box-Cox transformation to *y*_*i*_ to stabilize variance and improve normality (25). The optimal λ is determined to minimize deviations from normality and heteroscedasticity. This approach also reduces the effect of outlier counties with exceptionally high or low incidence rates. Then parameter estimates within the OLS framework remain unbiased and consistent, aligning with established epidemiologic methods and statistical modeling practices.

We also assessed multicollinearity among predictors. Each variable's variance inflation factor was maintained below 10. To achieve this, we omitted variables deemed less relevant. This rigorous variable selection enhances model parsimony and minimizes the risk of inflated standard errors, thereby supporting robust inference.

To handle potential heteroskedasticity and serial correlation in the error terms, we employ a Heteroskedasticity and Autocorrelation Consistent (HAC) covariance matrix estimator, we use it as a safeguard against potential deviations from homoscedasticity and serial independence (26). HAC would be implemented vcovHAC to adjust standard errors. This robust approach ensures that inference remains reliable in our county-level COVID-19 data, in line with established practices in spatial epidemiology (24, 26).

### 3.2 Spatial model

In county-level COVID-19 analyses, disease outbreaks often extend beyond administrative boundaries. Proximity fosters the transmission of SARS-CoV-2, leading to correlated residuals. This scenario violates OLS assumptions of independent error terms (27). Spatial econometric models address such concerns by incorporating structured dependence, either through the dependent variable or the error term. We adopt two prominent specifications: the Spatial Autoregressive Model (SAR) and the Spatial Error Model (SEM). Both rely on a spatial weights matrix *W*, constructed from county coordinates to reflect patterns of geographic adjacency.

To demonstrate the need for spatial models, consider the OLS regression:


(1) $$\begin{array}{l} y=X\beta +\varepsilon , \end{array}$$

where *y* is an *n*×1 vector of responses (in our case, the transformed COVID-19 incidence), *X* is an *n*×*k* matrix of explanatory variables (e.g., temperature, precipitation, population density, or socioeconomic factors), and ε denotes the error term.

The SAR model captures dependence through a spatial lag of the dependent variable:


(2) $$\begin{array}{l} y=\rho Wy+X\beta +\varepsilon , \end{array}$$

where *W* is an *n*×*n* spatial weights matrix, and ρ measures the strength and direction of spatial interaction (28). the matrix *W* was derived by imposing a threshold distance between county centroids, using their latitude and longitude. If ρ is positive and significant, counties with elevated COVID-19 incidence tend to be clustered near other counties with high incidence. This pattern aligns with the communicable disease process, where infection can spread through contiguous or adjacent populations. The parameter ρ thus represents how strongly the virus diffuses across county boundaries (29). In our county-level COVID-19 analyses, we construct the spatial weights matrix *W* as a row-standardized *k*-nearest-neighbors structure with *k* = 8, ensuring that every county has eight neighbors and thus an equal contribution to the spatial lag term—thereby avoiding the isolation of counties with few or no adjacencies under contiguity methods or the arbitrary cutoff and potential disconnects of fixed-distance thresholds; the choice of *k* = 8 reflects the typical range of U.S. county adjacencies, was validated through cross-validation of model fit and assessment of residual spatial autocorrelation via Moran's *I*, and strikes a balance between preserving global network connectivity and preventing excessive smoothing of localized effects (30). By modeling interactions among neighboring counties, we gain insights into how environmental monitoring, social factors, and population surveillance interact with local incidence.

Although the SAR model captures how COVID-19 spreads across nearby counties, it assumes that the spatial effect manifests through the dependent variable. However, unmeasured factors can also cluster geographically and produce correlated residuals (31). This possibility motivates the SEM, where spatial correlation stems from latent variables, policy choices, or other unobserved dynamics (27).

Formally, the SEM is written as:


(3) $$\begin{array}{l} y=X\beta +u,\;u=\lambda Wu+\varepsilon , \end{array}$$

where *y* denotes the transformed rate of COVID-19 cases per 10K, *X* is a matrix of covariates reflecting health, environment, or socioeconomic status, and *u* represents spatially dependent residuals. The term λ*Wu* captures hidden structures that diffuse across county borders via adjacency or proximity. As with the SAR formulation, *W* represents the spatial structure derived from latitude and longitude. However, in this model, the contagion effect is assumed to emerge through residual terms, rather than directly in the dependent variable. In the SEM model, *W* is constructed from latitude and longitude coordinates, ensuring that contiguous counties exert greater influence on each other.The coefficient λ indicates the magnitude of this unobserved spatial dependence (29). If it is large and significant, communities in proximity exhibit similar residuals, independent of explicit regressors (32).

By modeling the correlation in *u*, the SEM handles omitted risk factors and policy differences. Some counties might have localized testing, healthcare facility arrangements, or behavioral patterns that are not directly specified in *X*. When these features correlate with location, they induce clustering in the error term.

Recognizing that spatial models, such as SAR and SEM, capture local patterns of COVID-19 transmission, these approaches may still overlook broader institutional and administrative differences. State-level policy interventions, healthcare resource distributions, and legislative authorities can vary widely. This heterogeneity can shape testing coverage, vaccination programs, and hospital capacity, often transcending simple spatial adjacency (33, 34). Consequently, we introduce a hierarchical structure that incorporates state-specific effects, acknowledging that counties within the same state share regulatory frameworks and public health strategies (34).

In this hierarchical model, each county *i* belongs to state *j*, with *j* = 1, 2, …, *J*. A random-intercept formulation allows the model to account for unobserved variations at the state level:


(4) $$\begin{array}{l} y_{ij}=\alpha_{j}+\overset{K}{\underset{k=1}{\sum }}\beta_{k}x_{ij}+\varepsilon_{ij},\;\alpha_{j}=\alpha +\nu_{j},\;\nu_{j}\sim N\left(0,\sigma_{\nu }^{2}\right), \end{array}$$

where *y*_*ij*_ denotes the transformed COVID-19 incidence for county *i* in state *j*, *x*_*ij*_ is a vector of county-level covariates, and ν_*j*_ represents random intercepts capturing latent differences across states (35). Random intercepts α_*j*_ capture these statewide influences, while county-level predictors still explain within-state variation. Unlike pure spatial models that rely on geographic contiguity or distance metrics, this hierarchical specification emphasizes that governance structures, funding mechanisms, and policy mandates are state-based rather than merely spatially contiguous (36). For instance, two geographically distant counties within the same state might display similar patterns of disease management due to uniform mandates on mask usage or consistent resource allocations for testing.

### 3.3 Geographically weighted regression

Geographically Weighted Regression (GWR) extends traditional regression by allowing parameter estimates to vary across locations (30, 37). This spatial heterogeneity is crucial in public health contexts, where localized factors influence disease incidence.

At the core of GWR is a weighting scheme that emphasizes nearby observations. Each county *i* has coordinates (*u*_*i*_, *v*_*i*_) derived from its longitude and latitude. The model is then defined as:


(5) $$\begin{array}{l} y_{i}=\beta_{0}\left(u_{i},v_{i}\right)+\overset{K}{\underset{k=1}{\sum }}\beta_{k}\left(u_{i},v_{i}\right)x_{ik}+\varepsilon_{i}, \end{array}$$

where *y*_*i*_ is the transformed COVID-19 incidence for county *i*, and *x*_*ik*_ represents local variables such as Temperature, Precipitation, and other relevant epidemiologic factors (38). The terms β_*k*_(*u*_*i*_, *v*_*i*_) are location-specific parameters. By mapping these coefficients, researchers can explore how disease drivers vary spatially and gain insights into local vulnerability.

A kernel function governs how GWR assigns weights to observations based on their distance from county *i*. We adopt the bisquare kernel, which is defined by:


(6) $$w_{ij}=\{\begin{array}{ll} {\left[1-{\left(\frac{d_{ij}}{b}\right)}^{2}\right]}^{2}, & d_{ij}<b,\\ 0,otherwise, & \; \end{array}$$

where *d*_*ij*_ is the distance between counties *i* and *j*, and *b* is the bandwidth (39, 40). The bisquare function sharply reduces weights to zero beyond the distance *b*. This localized emphasis is often desirable in communicable disease studies, because it reflects the limited spatial range of interaction or environmental similarities (39). Counties outside the bisquare radius exert no influence on the coefficient estimates for county *i*.

Selecting an appropriate bandwidth *b* is essential for striking a balance between bias and variance. A large *b* smooths over broad regions, risking loss of local detail. A small *b* captures highly localized patterns, but may amplify random noise (39, 41). We use the corrected Akaike Information Criterion (AICc) to find the best *b* value. This procedure considers model complexity and fit, penalizing overparameterization (42). In our analysis, the AICc-based search yields a bandwidth of 9.012423, indicating that counties within this radius meaningfully affect each other's parameter estimates. This distance threshold captures mid-range spatial dependence in COVID-19 data, reflecting factors such as shared healthcare resources or regional climatic conditions.

The GWR fitting process then solves, for each location (*u*_*i*_, *v*_*i*_):


(7) $$\begin{array}{l} \hat{\beta }\left(u_{i},v_{i}\right)=\underset{\beta }{\arg\min}\overset{n}{\underset{j=1}{\sum }}w_{ij}{\left(y_{j}-\beta^{⊤}x_{j}\right)}^{2}, \end{array}$$

where *w*_*ij*_ is calculated via the bisquare kernel, and *x*_*j*_ is the vector of covariates for county *j* (37). We implement this in our model to ensure that the bandwidth and kernel choice remain consistent. The algorithm produces a distinct set of coefficients β_0_(*u*_*i*_, *v*_*i*_), …, β_*K*_(*u*_*i*_, *v*_*i*_) for each county. These local estimates help detect spatial variations in key predictors, such as Temperature or Precipitation, and measure how each variable's influence on COVID-19 incidence changes from one region to another.

### 3.4 Framework

Our framework unites OLS, SAR models, SEM, hierarchical modeling, and GWR to address multifaceted challenges in epidemiological methods and data interpretation. We start with OLS to establish a baseline for assessing statistical associations. However, OLS ignores contagion and can underestimate standard errors if spatial heterogeneity exists. By incorporating spatial analysis through SAR, we capture direct influences of neighboring counties on infection rates, thus clarifying regional diffusion processes (29). If the Moran's *I* test on the SAR model remains significant, it indicates that some spatial effects have not been captured, thereby justifying the application of SEM. SEM further accounts for unmeasured spatial factors that propagate disease risk, offering deeper insights when certain variables are difficult to measure or remain unknown (31).

Even these spatial models may overlook hierarchical differences among states, such as disparities in healthcare resources, legislative authority, or public health policies (33, 34). Our hierarchical model acknowledges that counties within the same state share overarching regulatory frameworks, leading to more accurate model coefficients. This approach ensures that administrative heterogeneity is considered alongside spatial adjacency. Lastly, GWR reveals local variations in infection drivers by estimating location-specific coefficients (39, 40). This local modeling strategy shows how environmental parameters, such as precipitation or temperature, affect COVID-19 incidence in distinct regions. By highlighting geographic variations in disease determinants, GWR aids public health practitioners in tailoring interventions and resource allocations (37, 38).

**Figure 3:**
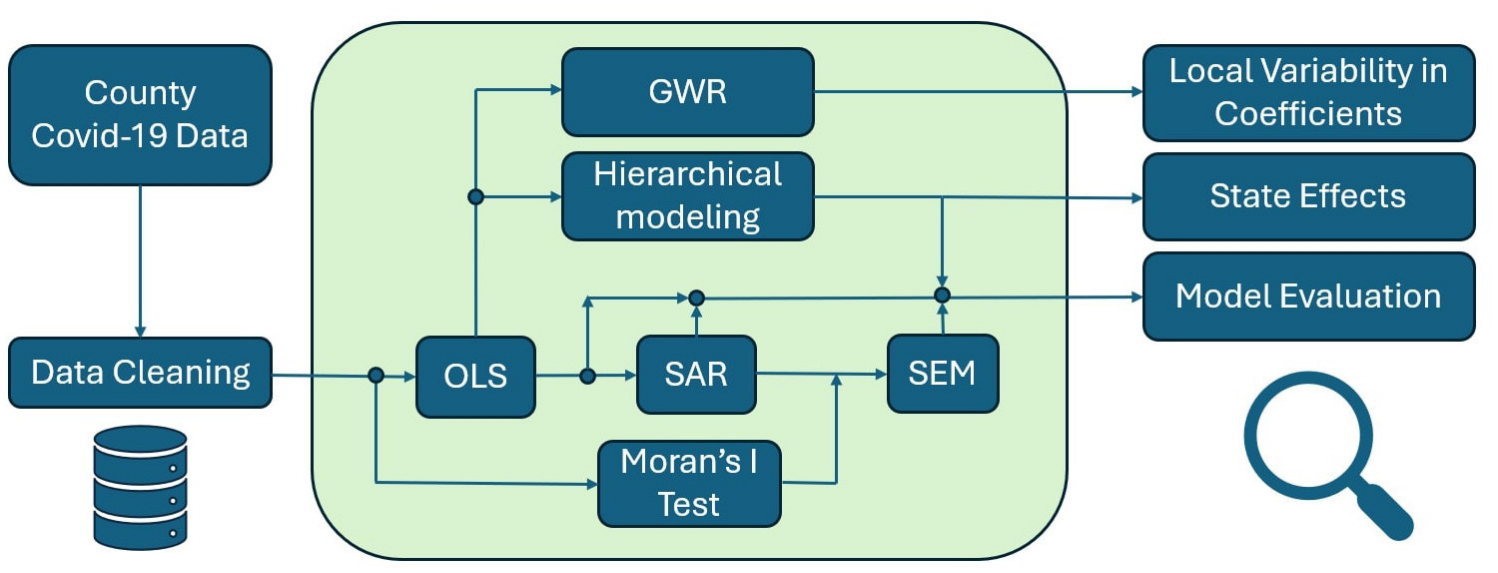
A flowchart summarizing the analytical procedure: starting from county-level COVID-19 data, the process moves through data cleaning and OLS, followed by spatial tests and models (Moran's *I*, SAR, SEM), with parallel paths for hierarchical modeling and GWR to assess state policy effects and local variability.

In combination, these models deliver a comprehensive statistical framework (see Figure 3). Each component addresses different aspects of spatial heterogeneity, hierarchical clustering, and local non-stationarity. By systematically applying OLS, SAR, SEM, hierarchical modeling, and GWR, we move beyond naive regression analyses. This strategy produces robust inferences, improves predictive accuracy, and supports evidence-based decisions in health and medicine.

## 4 Result

The core response variable consists of county-level confirmed cases, measured as cases per 10k population. To ensure that the linear regression assumptions hold, we first evaluate the distribution of this outcome variable. The original data exhibits a pronounced skew, which can violate normality assumptions necessary for standard OLS estimation. Hence, we apply a Box-Cox transformation with a coefficient of 0.263. This transformation successfully reduces the skewness of the dependent variable to 0.0577, moving it closer to a normal distribution and strengthening the validity of subsequent linear models.

Interpreting coefficients after transformation is less direct than in untransformed models. A one-unit increase in a predictor changes the transformed outcome *y*^(0.263)^ by β, not the original case rate. To express the effect in public health terms (e.g., cases per 10,000 population), we back-transform using:


(8) $$\begin{array}{l} ŷ={\left(0.263\cdot {ŷ}^{\left(0.263\right)}+1\right)}^{1/0.263} \end{array}$$

We then compute the difference in predicted values after increasing the predictor by one unit:


(9) $$\begin{array}{l} \Delta y={\left(0.263\cdot \left({ŷ}^{\left(0.263\right)}+\beta \right)+1\right)}^{1/0.263}-{\left(0.263\cdot {ŷ}^{\left(0.263\right)}+1\right)}^{1/0.263} \end{array}$$

This gives the change in expected cases per 10,000 population. While not as immediately interpretable as models on the original scale, this approach still yields clear, policy-relevant insights.

In parallel with the transformation, we explore the presence of spatial dependence in the data. Using Moran's *I* test, we find that the cases per 10k exhibit significant spatial clustering (Moran's *I* = 0.5944, *p*-value < 2.2e-16). Such strong spatial autocorrelation indicates that the infection rates are not randomly distributed across counties (22). High spatial clustering can violate the independence assumptions implicit in basic OLS, advanced spatial models become relevant to adequately capture these Epidemiology and Public Health processes (36, 37).

### 4.1 Model development and comparison

We construct a series of regression analyses by relating the transformed case count variable (*cases_per_10k_transformed*) to a range of explanatory factors, including meteorological variables, demographic characteristics, mobility indicators, and measures of population density, socio-educational status, and healthcare capacity. Table 2 outlines the coefficients, significance, and direction of effects for each predictor. The regression analysis results indicate substantial differences in model performance between the OLS model and the spatial models. Table 3 summarizes the goodness-of-fit metrics for the four global models.

**Table 2:** Comparisons of four models (OLS, SAR, SEM, multi-level).

**Variable**	**OLS**		**SAR**		**SEM**		**Multi-level**	
	**Coef**	*p* **-value**	**Coef**	*p* **-value**	**Coef**	*p* **-value**	**Coef**	*p* **-value**
temp	0.104	< 0.01	0.015	0.06	0.098	< 0.01	0.120	< 0.01
precip	0.021	< 0.01	0.006	0.02	0.011	0.04	0.005	0.15
young	0.113	< 0.01	0.059	< 0.01	0.035	0.04	0.030	0.11
old	–0.064	< 0.01	–0.068	< 0.01	–0.113	< 0.01	–0.129	< 0.01
SD_total	–0.037	0.30	–0.110	< 0.01	–0.139	< 0.01	–0.106	< 0.01
SD_travel	–0.054	0.10	0.058	0.04	0.099	< 0.01	0.014	0.65
daily_test	–1.6e–05	< 0.01	–7.6e–06	0.02	–2.e–06	0.75	–6.6e–06	0.69
pop_dens	4.e–05	0.07	7.4e–06	0.69	–3.3e–05	0.12	4e–05	0.04
female_pct	–15.9	< 0.01	–11.906	< 0.01	–8.85	< 0.01	–7.086	< 0.01
area	7.7e–05	0.02	6.2e–05	0.03	1.3e–04	< 0.01	9.1e–05	0.01
vent_cap	100.7	0.39	168.9	0.08	150.56	0.10	66.6	0.52
less_HS	0.175	< 0.01	0.126	< 0.01	0.166	< 0.01	0.171	< 0.01
some_coll	0.065	< 0.01	0.039	< 0.01	0.038	< 0.01	0.030	< 0.01
tot_coll_pop	0.215	0.10	0.178	0.09	0.124	0.21	0.223	0.05
diabetes_pct	0.020	0.10	0.005	0.60	3.9e–04	0.97	0.012	0.25
relig_cong	0.030	< 0.01	0.015	< 0.01	0.013	< 0.01	0.020	< 0.01
pol_party	–0.863	< 0.01	–0.381	< 0.01	–0.569	< 0.01	–0.731	0.08
ap_dist	–0.005	< 0.01	–0.002	< 0.01	–0.005	< 0.01	–0.002	< 0.01
pass_load	–0.003	0.44	–0.004	0.22	–0.005	0.15	–0.004	0.27
meat_plant	0.020	< 0.01	0.015	< 0.01	0.010	0.01	0.014	< 0.01
insured_pct	0.052	< 0.01	0.020	0.04	6.9e–04	0.96	–4.1e–04	0.98
gdp_pc	–1.03e–04	< 0.01	–1.12e–04	< 0.01	–9.3e–05	< 0.01	–1.1e–04	< 0.01
immig_stud	–2.133	0.46	0.199	0.93	0.905	0.68	–0.672	0.79
med_income	–5.6e–07	0.91	–8.5e–06	0.04	–1.3e–05	0.01	6.9e–06	0.17
hosp_beds	35.1	< 0.01	17.466	0.05	14.661	0.07	18.680	0.04
smokers_pct	0.031	0.09	0.030	0.05	0.022	0.22	0.024	0.25

**Table 3:** Model performance metrics.

**Model**	***R* ^2^**	**RMSE**
OLS	0.4849	2.0891
SAR	0.6501	1.7454
SEM	0.6846	1.6420
Multi-level (conditional *R*^2^)	0.6615	1.8412

The OLS model exhibits moderate goodness-of-fit, with HAC-adjusted coefficients ensuring compliance with standard assumptions. In comparison, the SAR model, which incorporates a spatially lagged dependent variable, yields a notably higher R^2^ and a statistically significant SAR coefficient, indicating an improved capture of variance in COVID-19 case rates. The SAR model increased the R^2^ from 48% to 65%, demonstrating its effectiveness in capturing the direct influence of neighboring counties on infection rates. However, a Moran's *I* test on the SAR residuals yielded a significant *p*-value (*p* = 0.002745), indicating that substantial spatial autocorrelation remains unaddressed. This finding justified the subsequent use of a SEM to capture the remaining unobserved spatial effects. The SEM model produced a non-significant Moran's *I* (*p* = 0.97), and further improvements in R^2^ and RMSE metrics confirmed that, although more computationally intensive, SEM provides a more robust and accurate framework for modeling spatial dependencies in the data. The SEM approach demonstrates an additional improvement in R^2^, and its spatial error coefficient is statistically significant. This progression from SAR to SEM confirms that incorporating unobserved, spatially structured factors leads to a more robust model. Both spatial models demonstrate that incorporating spatial autocorrelation which is a key aspect of spatial analysis in epidemiology, yields more accurate predictions and better fit than a standard OLS regression.

The multilevel model offered another perspective by accounting for hierarchical structure in the data. The multilevel model's fixed effects yielded a marginal $R_{m}^{2}=0.4810$, very similar to the OLS *R*^2^. However, the conditional *R*^2^ accounting for both fixed and random effects rose to 66.15%. Thus, incorporating random intercepts for region dramatically improved the variance explained, on par with the spatial models. In practical terms, about 0.6615 − 0.4810 ≈ 0.1805 (18%) additional variance was explained by adding higher-level effects. This underscores that a substantial portion of the variability in COVID-19 case rates is attributable to differences between state, beyond what is captured by observed county-level covariates. The multilevel model's performance is similar to SAR/SEM model, demonstrating that accounting for hierarchical clustering can rival the explanatory power of explicit spatial lag or error terms.

It should be noted that the computation of *R*^2^ differs among OLS, SEM, SAR, and multilevel models. Each method quantifies explained variance in a distinct manner. OLS employs a simple ratio of explained to total variance. Spatial models adjust the measure to incorporate spatial dependence. Multilevel models yield marginal and conditional *R*^2^ indices. This variability limits direct comparison of model fit (43). Caution is warranted when interpreting these metrics in epidemiologic research (35, 44). Nonetheless, comparing the *R*^2^ from spatial models with the classical OLS *R*^2^ remains a common heuristic for assessing the added value of spatial dependence in public-health and spatial-econometric studies (29). To provide a scale-invariant check, we also compute the RMSE. The RMSE results corroborate the *R*^2^ ordering: both SEM and SAR models outperform OLS, and the multilevel also achieves the relative low RMSE, underscoring its predictive accuracy in this context.

### 4.2 Feature analysis

In our analysis, several additional predictor variables demonstrated significant associations with COVID-19 case rates, offering deeper insights into the pandemic's dynamics. This study shows that temperature exerts a significant effect on COVID-19 incidence across models, suggesting a critical role in modulating viral viability and social behavior (45). Conversely, precipitation's effect is inconsistent across models, with its statistical significance markedly attenuated in spatial specifications compared to its robust performance in the OLS framework. In spatial models, the weakened precipitation coefficient suggests that spatial autocorrelation and unobserved heterogeneity may dilute its apparent impact (43, 46).

The proportion of young individuals consistently exhibited a positive association with COVID-19 case rates across multiple models. This suggests that younger populations may have higher transmission rates, potentially due to increased social interactions or lower adherence to preventive measures. Conversely, the proportion of older adults showed a negative association with case rates, indicating that areas with higher older populations experienced lower transmission rates. This could be attributed to stricter adherence to preventive measures among older adults or targeted public health interventions aimed at protecting vulnerable populations.

The percentage of residents without a high school diploma was positively associated with COVID-19 case rates. This underscores the role of educational attainment in health literacy and adherence to preventive measures. Individuals with lower educational levels may have limited access to accurate health information, leading to behaviors that increase transmission risk.

The presence of meat processing plants was positively associated with case rates. These facilities have been identified as high-risk settings for COVID-19 outbreaks due to close working conditions and challenges in implementing preventive measures (47). Neither the percentage of smokers nor the percentage of diabetes showed significant associations with COVID-19 transmission rates in our models. Our model indicates that their influence on viral spread appears limited. This may indicate that these factors primarily affect disease severity rather than transmission dynamics. Proximity to airports was negatively associated with case rates, indicating that areas farther from major transportation hubs experienced lower transmission. This could be due to reduced travel-related spread in these regions.

Across all four models, temperature shows a consistent positive link with COVID-19 incidence in every model. This stability holds after we correct for spatial dependence and add state-level random effects. In contrast, precipitation looks important only in the naïve OLS and SAR. Its influence disappears once spatial error or hierarchical structure is considered. Social-distancing grades tell the opposite story. They become strongly protective in the three spatial or multilevel models, even though they were silent in OLS. The share of young adults remains a risk factor, yet its effect shrinks when state heterogeneity is isolated. Political affiliation follows a similar pattern. It is significant in SAR and SEM but fades in the multilevel model, suggesting that partisan signals act mainly through state policy rather than local ideology. These shifts matter. Spatial and hierarchical methods not only tighten statistical fit; they reveal which drivers act at county vs. state scales. Ignoring those scales can mask true climate effects, inflate behavioral signals, and blur policy guidance. Short, scale-aware models therefore give clearer insights for public health planning.

The analysis of these additional predictor variables provides insights into the multifaceted factors influencing COVID-19 case rates. Demographic characteristics, socioeconomic status, environmental settings, and health behaviors all play significant roles in shaping transmission dynamics. Understanding these associations is crucial for tailoring public health interventions and resource allocation to effectively mitigate the spread of COVID-19.

### 4.3 GWR analysis

Beyond the global regression models, a GWR was applied to explore spatial non-stationarity in the relationships between COVID-19 case rates and select predictors. We focused on environmental variables (temperature and precipitation) in the GWR analysis, given their potential spatially varying effects on virus transmission dynamics.

**Figure 4:**
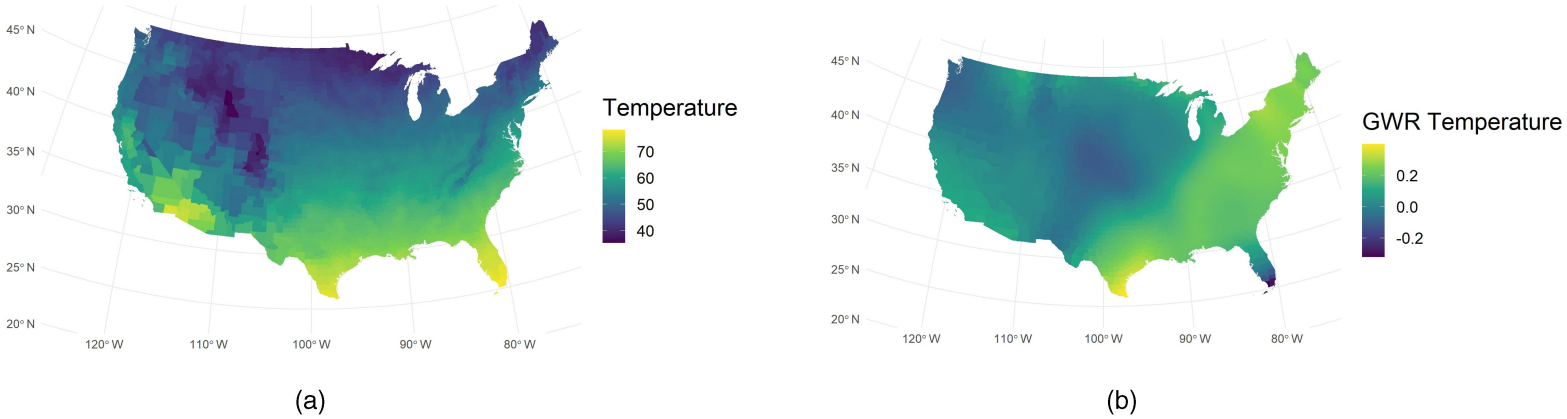
Comparison of observed and GWR-derived temperature. **(a)** Observed temperature. **(b)** GWR temperature.

Figure 4a illustrates the spatial distribution of temperature across the United States, with higher temperatures observed in lower-latitude regions. Figure 4b shows the local GWR coefficients varied substantially by region. In southeastern and northeastern counties, coefficients reached around 0.2, indicating a strong positive association with COVID-19 incidence. In contrast, coefficients in many midwestern counties were near zero, suggesting a limited effect of temperature in those areas. These results imply that in regions with higher ambient temperatures, such as the Southeast and Northeast, temperature may more strongly modulate disease spread—possibly by influencing human behavior and outdoor activities—than in cooler, midwestern areas.

**Figure 5:**
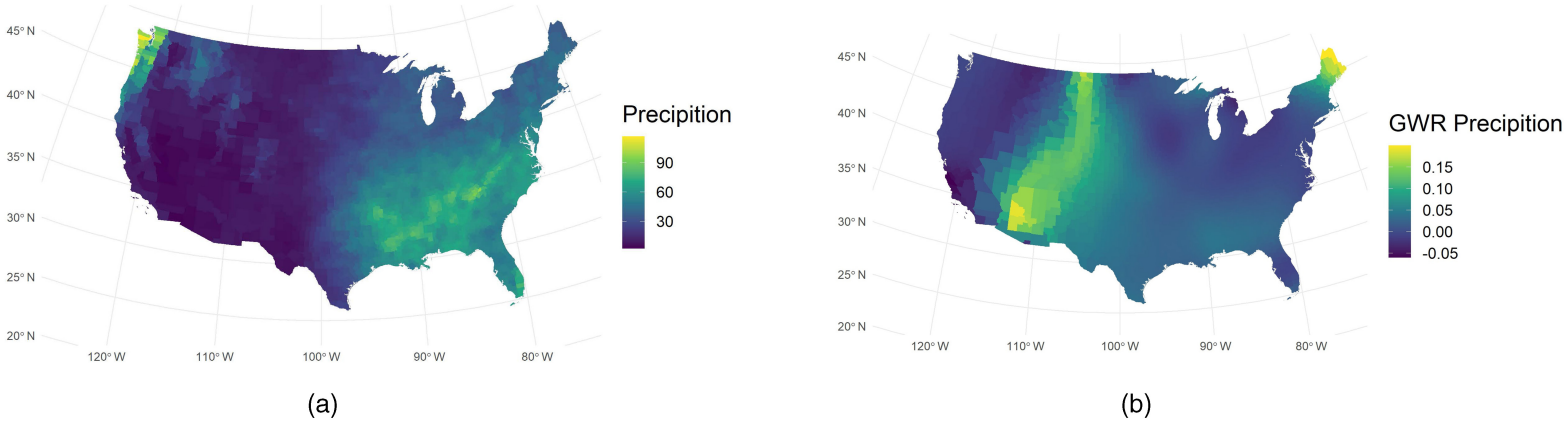
Comparison of observed and GWR-derived precipitation. **(a)** Observed precipitation. **(b)** GWR precipitation.

Also, we use GWR analysis yielded revealing insights into the relationship between precipitation and COVID-19 spread. Figure 5b indicates that the GWR-derived sensitivity to precipitation peaks near 110° W. This peak is strikingly inverse to the actual precipitation distribution shown in Figure 5a. Such an inverse pattern is consistent with prior investigations linking meteorological variables to viral transmission (7), which is that low humidity in arid regions can significantly enhance the transmission of COVID-19 (7). Moreover, GWR precipitation remains high in the northeastern United States during periods of low incidence. This phenomenon may imply that low case rates do not always coincide with reduced environmental exposure to precipitation or other weather factors. Instead, our model points to a possible interplay of epidemiologic factors, such as population density and mobility, with local weather conditions.

The spatial variation we observe underscores the importance of Spatial Analysis in public health research. GWR isolates local effects that would otherwise remain obscured by conventional Regression Analysis techniques. The significance maps for the temperature and precipitation coefficients are presented in Figure 7 of the Supplementary material.

## 5 Discussion and conclusion

Our findings reveal that aggregated county-level data offer powerful insights into COVID-19 transmission dynamics. By combining daily observations, we captured long-term patterns and mitigated short-term noise. Our results underscore the importance of spatial heterogeneity. Counties with similar demographic traits can exhibit divergent infection rates if regional factors differ. We observed significant clustering based on Local Moran's *I*, confirming that infection processes follow geographic boundaries in ways that transcend administrative units (22, 23).

We employed four distinct models for this analysis, and the spatial model achieved the strongest performance. However, most risk factors for COVID-19 aligned with previous findings (48), thus reinforcing mutual validation. One key variable was the Social Distancing grade. Counties with higher grades demonstrated better Infection Control of Covid-19 transmission, as shown in Figure 2c. In three spatial models, its coefficients were negative with significant *p*-values, implying that rigorous disease outbreaks control correlates with reduced spread. This outcome also matched the pattern for age structure. Counties with a larger young adult proportion exhibited higher COVID-19 incidence, possibly because youthful populations engage in more social contact. In contrast, counties with more older adults showed lower incidence, likely due to stricter adherence to protective measures (49). Educational attainment also contributed to mitigating Transmission, as higher education often increases awareness of Public Health guidelines. Conversely, the presence of meat plants can accelerate spread, partly because Ventilation challenges increase risk factors for COVID-19 (50).

On the other hand, temperature and precipitation displayed an unexpected positive association with COVID-19 in the continental United States (Figure 2b). Prior work often suggests a negative effect, where lower temperature and humidity boost viral activity (7). Our cross-sectional data may fail to capture temporal variations, especially since southern regions had higher COVID-19 rates than northern areas. This phenomenon could reflect other unmeasured factors. A more robust approach might involve tracking seasonal changes within each county or state, correlating local temperature and precipitation with COVID-19 test data. That approach may clarify whether such climate variables truly exhibit a positive relationship, or if they simply coincide with geographic patterns.

Southeastern counties fall into high-high clusters (based on Moran's *I*), suggesting regional factors—such as relaxed containment measures or high indoor congregation may amplify transmission (23, 51). In contrast, many Northeastern counties form low-low clusters, possibly reflecting stricter public health interventions and a collective response informed by early pandemic surges. This sharp disparity implies that meteorological conditions alone cannot explain local COVID-19 dynamics. Instead, community mitigation behaviors, policy enforcement, and historical disease exposure likely mediate the observed temperature sensitivity (52). Southeastern states face higher risk due to a combination of elevated ambient temperature and fewer preventive measures, whereas Northeastern regions benefit from aggressive social distancing, targeted resource allocation, and entrenched healthcare infrastructure. These findings underscore the importance of examining not just climate variables, but also the broader social and policy context that shapes disease incidence and progression.

GWR-derived sensitivity to precipitation peaks near 110° W, contrasting sharply with the actual rainfall distribution. We posit that dryness may prolong aerosol persistence, thus amplifying viral transmission (7, 51). Arid regions often lack adequate ventilation and well-enforced preventive strategies, which can further intensify pathogen circulation indoors (53). Evidence also suggests low humidity can accelerate respiratory disease spread, highlighting the joint influence of climate and public health measures (7).

The multilevel analysis showed that political party, precipitation, and young people proportion lost significance once state-level variation was introduced, even though they had been significant in a pure spatial framework (see Table 2). This outcome suggests that state policies or macro-level conditions overshadow county-level effects. Politics and Public Policy can drive far-reaching changes in healthcare access, education, and resource distribution, explaining why political affiliation lost direct impact under random intercepts. Likewise, Precipitation might reflect broader environmental or funding patterns that are better modeled at higher tiers. Young Adult attributes vary more by statewide initiatives than by local differences. This approach helps partition the variance between states and counties, revealing that local variables can appear significant in spatial models but become overshadowed when broader legislative frameworks are accounted for (54). In terms of fit, R-square and RMSE improved notably compared to standard OLS, though differences from SAR or SEM were modest. Future analyses may require refined data on State Government interventions, such as healthcare subsidies or demographic programs. These findings align with Health Services Research that emphasizes the interplay between state mandates and local demographics. Such insights underscore the importance of incorporating multiple administrative layers to advance robust public health strategies.

These results lead to some clear policy actions. First, position mobile vaccination and booster clinics in GWR-identified hotspots—counties that combine high temperature, low humidity, and poor distancing—to curb cluster growth (55). Second, require state health agencies to operate a real-time dashboard that tracks county-level distancing grades, testing volume, and hospital load, triggering rapid resource shifts whenever thresholds are breached (56). Third, enforce seasonal ventilation upgrades and routine screening in meat plants, airports, and other crowded workplaces within arid regions, where warm, dry air prolongs aerosol persistence (50). These targeted, scale-aware measures translate our statistical evidence into actionable public health practice.

In conclusion, this work demonstrates that a multi-method strategy can deliver robust, nuanced conclusions for public health research. Aggregation into county-level cross-sectional data reduces day-to-day noise. Our framework begin with an OLS baseline, we quickly observe that spatial dependence biases standard errors, prompting the use of SAR and SEM that better account for near-county interactions (31). Spatial econometric models reveal the contagious nature of infection. Hierarchical models highlight state-level heterogeneity. GWR documents local variability in weather effects. Together, these findings underscore the critical importance of tailored interventions, especially when addressing diverse populations and regions. Policy makers should integrate spatial, demographic, and hierarchical insights to allocate resources effectively. Future studies may refine this framework by incorporating real-time data, testing intensities, or contact-tracing metrics. This layered and statistics-based approach paves the way for evidence-based decision-making and more precise control of emerging pandemics.

## 6 Limitations and future work

In our framework, social distance total grade, social distancing encounters grade, and social distancing travel distance grade, the mean values were calculated across the study period. Static county-level attributes (e.g., demographic, socioeconomic, and health system indicators) remained unchanged. Each county serves as a single aggregated observation, reducing short-term noise and random measurement error, highlighting long-term trends, and aligning analyses with policy-relevant administrative boundaries. This aggregation also mitigates temporal autocorrelation, enabling a clearer focus on spatial heterogeneity.

However, reliance on aggregated county-level summaries can obscure temporal variability and introduce ecological fallacy, limiting the ability to detect short-lived spikes or lagged effects that are critical for timely intervention efforts (57, 58). Our next work will focus on adopting a fine-grained time-series framework to capture dynamic social distancing fluctuations at the county level and address the biases introduced by aggregation.

Additionally, our analysis omits the effects of COVID-19 vaccination campaigns and the dynamic influence of emerging SARS-CoV-2 variants—potentially biasing estimates of social distancing efficacy (55, 59). In future work, we will update our county-level dataset with dynamic vaccination coverage and variant prevalence metrics and leverage fine-grained time-series analyses to address these shortcomings.

## Data Availability

The original contributions presented in the study are included in the article/supplementary material, further inquiries can be directed to the corresponding author.
