# Supplementary material for: A framework for modeling county-level COVID-19 transmission
Source: Front Public Health. 2025 Aug 5;13:1608360. doi: 10.3389/fpubh.2025.1608360 (PMC12361220; doi:10.3389/fpubh.2025.1608360)
Supplement: Supplementary file 1 [file Presentation_1.pdf]

Supplementary Material

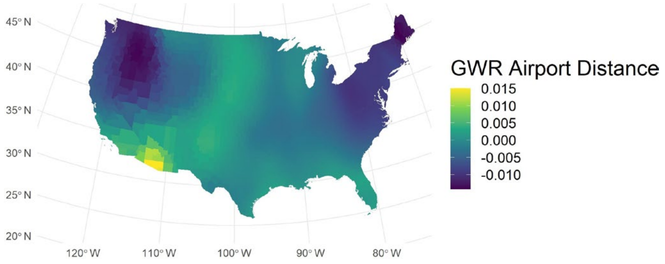

S1(a)

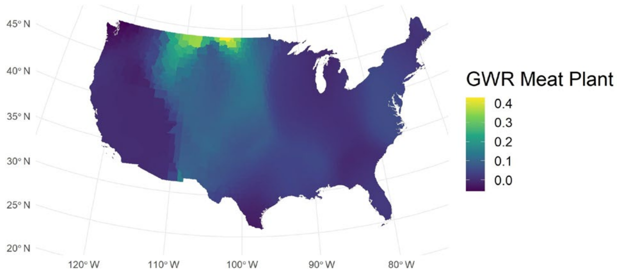

S1(b)

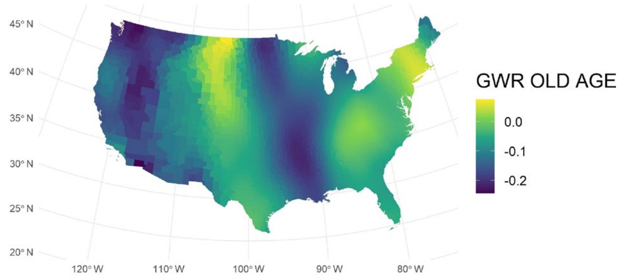

S1(c)

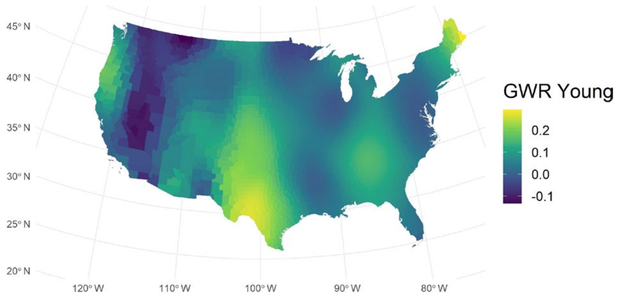

S1(d)

Figure S1: GWR results for selected important and significant variables.

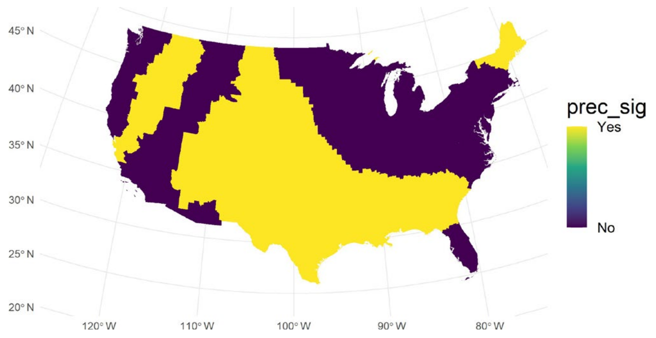

S2(a)

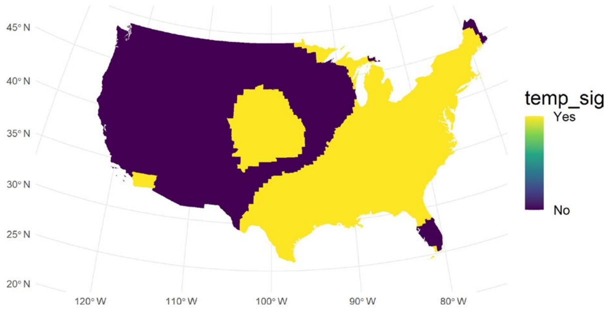

S2(b)

Figure S2: GWR significance maps for temperature and precipitation coefficients ($p < 0.05$).

We present the GWR results for selected variables that were identified as both important and statistically significant in the analysis. The following figures illustrate the spatial variations in their estimated coefficients.

We also generated GWR significance maps for the temperature and precipitation coefficients to identify where these climatic effects reach statistical significance. Figure S2(a) illustrates the significance pattern for the precipitation coefficient. Likewise, Figure S2(b) presents the spatial distribution of significance for the temperature coefficient ($p < 0.05$), revealing that its influence is strongest and statistically reliable in the northern and southeast counties, whereas east regions exhibit non-significant estimates. These maps underscore the spatial heterogeneity not only in the magnitude but also in the reliability of climatic drivers across our study area.
